# Mechanical removal of surface residues on graphene for TEM characterizations

**DOI:** 10.1186/s42649-020-00048-1

**Published:** 2020-11-30

**Authors:** Dong-Gyu Kim, Sol Lee, Kwanpyo Kim

**Affiliations:** Department of Physics, Yonsei University, Seoul, South Korea

**Keywords:** Atomic force microscopy, Mechanical cleaning of 2D crystals, PDMS residues, Mechanical transfer

## Abstract

Contamination on two-dimensional (2D) crystal surfaces poses serious limitations on fundamental studies and applications of 2D crystals. Surface residues induce uncontrolled doping and charge carrier scattering in 2D crystals, and trapped residues in mechanically assembled 2D vertical heterostructures often hinder coupling between stacked layers. Developing a process that can reduce the surface residues on 2D crystals is important. In this study, we explored the use of atomic force microscopy (AFM) to remove surface residues from 2D crystals. Using various transmission electron microscopy (TEM) investigations, we confirmed that surface residues on graphene samples can be effectively removed via contact-mode AFM scanning. The mechanical cleaning process dramatically increases the residue-free areas, where high-resolution imaging of graphene layers can be obtained. We believe that our mechanical cleaning process can be utilized to prepare high-quality 2D crystal samples with minimum surface residues.

## Introduction

Two-dimensional (2D) crystals have attracted widespread attention in recent years due to their emerging properties and potential applications in various fields (Butler et al. 2013; Fiori et al. 2014). Various physical, chemical, and electrical properties of 2D crystals are distinct from their bulk counterparts due to quantum confinement in few-atom-thick systems (Butler et al. 2013; Fiori et al. 2014). Moreover, 2D heterostructures prepared by assembling various 2D crystals in the lateral or vertical directions serve as new platforms for various investigations and applications (Geim and Grigorieva 2013). In these systems, the surface quality of 2D crystals, including the degree of residual surface contamination, is important, and surface contamination on 2D crystals often poses serious limitations on fundamental studies and applications (Chen et al. 2016; Dean et al. 2010). For example, surface residues on 2D crystals induce uncontrolled doping, charge carrier scattering, and trapped residues in mechanically assembled 2D vertical heterostructures (Chen et al. 2016; Dean et al. 2010). Therefore, developing a process that can reduce the surface residues on 2D crystals is vital.

Transmission electron microscopy (TEM) is an important characterization tool to investigate the structural quality of 2D crystals, especially their surface quality (Meyer et al. 2008; Rummeli et al. 2019). Previous TEM investigations revealed that 2D crystals prepared using various sample preparation processes suffer from surface contamination (Alemán et al. 2010; Lin et al. 2012). Surface contamination includes hydrocarbon, polymer residues, and under-etched metal residues (Alemán et al. 2010; Lin et al. 2012). Preparing residue-free samples is essential for reliable atomic-resolution TEM research. Previous studies indicated that plasma treatment, annealing at high temperature, and mechanical cleaning can remove surface residues induced by sample preparation methods (Goossens et al. 2012; Lim et al. 2012; Lin et al. 2012; Lindvall et al. 2012; Tripathi et al. 2017). However, the plasma treatment or high-temperature annealing process have potential to introduce defects in 2D crystals, and therefore require careful optimization. Moreover, various 2D layered crystals with high surface reactivity are not generally compatible with these process. On the other hand, the mechanical cleaning process has potential to remove residues on wide range of 2D crystals while minimizing the introduction of defects. In spite of its advantages, the effect of the mechanical cleaning process was rarely confirmed with TEM characterizations.

In this study, we explored the potential of using atomic force microscopy (AFM) to remove surface residues from 2D crystals (Goossens et al. 2012; Jain et al. 2018; Lindvall et al. 2012; Rosenberger et al. 2018; Schweizer et al. 2020). Using various TEM investigations, we confirmed that polydimethylsiloxane (PDMS) residues on graphene samples are effectively removed by contact-mode AFM sweeping. The mechanical cleaning process increases the residue-free area, where high-resolution imaging of graphene layers is feasible. The mechanical cleaning process is fairly simple and can be applied to prepare TEM specimens with other 2D materials. We posit that our mechanical cleaning process can be utilized to prepare high-quality 2D crystal samples with minimum surface residues.

## Materials and methods

### Sample preparation

We mechanically exfoliated graphene onto PDMS film. A silicon base and curing agent ratio of 10:1 was used to fabricate the PDMS film. The film was placed in a vacuum chamber for 1 h and heated using a hotplate at 60 °C for 1 h and 30 min. A graphene flake on PDMS identified by an optical microscope was transferred to a holey Si_3_N_4_ TEM grid by stamping. All the mechanical exfoliation and transfer processes were conducted at room temperature under ambient conditions. The TEM sample was annealed on the hotplate at 200 °C for 1 h with activated carbon (Algara-Siller et al. 2014) prior to AFM.

### Mechanical cleaning

We used AFM (Model XE-7, Parks Systems) in the non-contact mode (NCHR cantilever, with a 0.5 Hz scan rate and scan pixel number of 256) to obtain topographic images prior to the cleaning process. The mechanical cleaning was conducted by contact-mode AFM scanning with a scanning velocity of 0.3 μm/s, scan pixel number of 512, and vertical force of 3000 nN. After the cleaning process, topographic images were reobtained with the non-contact mode.

### TEM characterizations

TEM imaging, scanning transmission electron microscopy (STEM) imaging, and energy dispersive X-ray spectroscopy (EDX) mapping were conducted with a double Cs-corrected JEOL JEM-ARM200F operated at 80 kV.

## Results and discussion

**Fig. 1:**
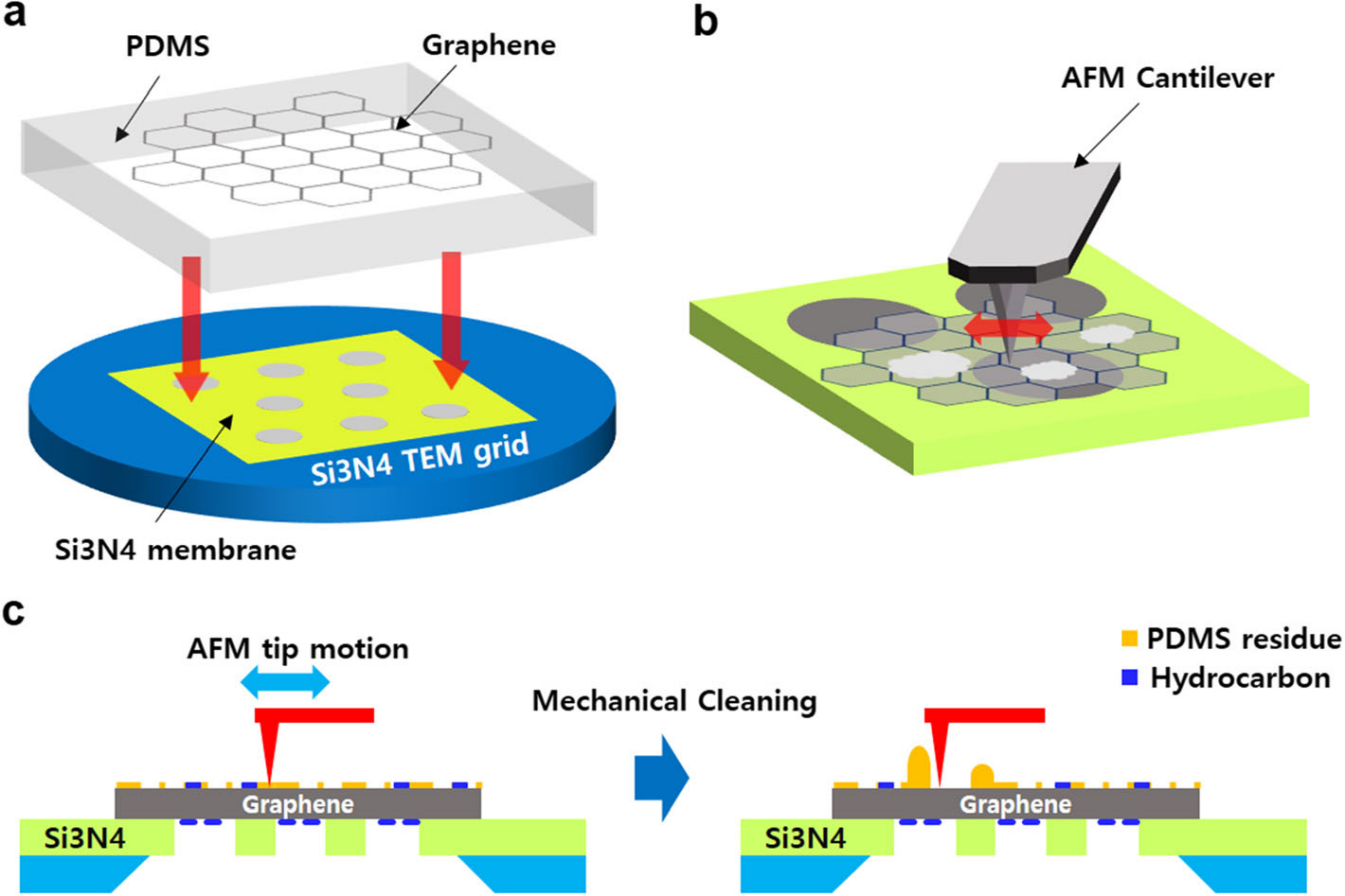
Schematics of the AFM-based mechanical cleaning process of a TEM sample. **a** Schematic illustration of the sample fabrication of graphene on a Si_3_N_4_ TEM grid membrane by PDMS-based stamping. **b** Schematic illustration of AFM-based cleaning of PDMS residue on a graphene TEM grid. **c** Side-view schematic of AFM-based cleaning. PDMS residues on the graphene’s top surface are removed by AFM-based scanning

Schematics of the TEM sample preparation and AFM-based mechanical cleaning processes are shown in Fig. 1. In this study, graphene served as a benchmark sample and other 2D crystals can be potentially processed using a similar sample preparation procedure. We first prepared graphene samples on PDMS film by mechanical exfoliation (Fig. 1a). The exfoliated graphene samples (~ 5 layers) were identified with an optical microscope and subsequently transferred to a holey Si_3_N_4_ membrane TEM grid by stamping (Fig. 1a). The stamping process mediated by PDMS film is simple to perform and was widely adapted in many prior studies (Dean et al. 2010; Jain et al. 2018; Rosenberger et al. 2018). In particular, the PDMS-based stamping process has been primarily used to fabricate 2D vertical heterostructures (Dean et al. 2010). However, the surface of 2D crystals prepared by mechanical transfer can suffer from PDMS residues and requires special attention, especially for surface-sensitive studies. After we prepared a TEM sample, we performed AFM contact-mode scanning on the TEM grid. We anticipated that surface residues on graphene could be swept away resulting in a residue-free surface (Fig. 1c).

**Fig. 2:**
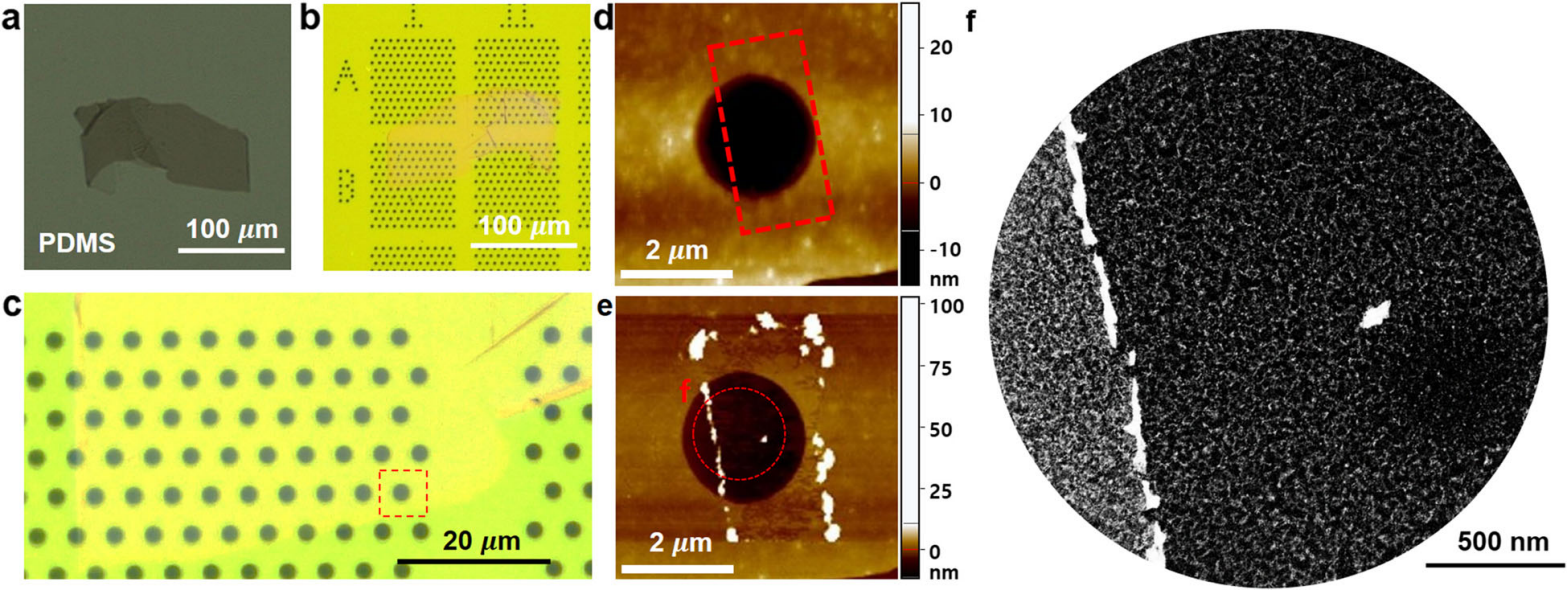
AFM and TEM investigation of mechanical cleaning of graphene surface residues. **a** Optical microscope image of a graphene flake exfoliated on the PDMS film. **b** Graphene flake transferred on a holey Si_3_N_4_ membrane TEM grid. **c** Close-up optical image. The hole in the dashed square was used for AFM scanning shown in panel (**d**). **d** AFM topography image of the as-prepared graphene near a hole in the Si_3_N_4_ membrane. The red dashed box indicates the AFM sweeping area. **e** AFM topography image obtained after the mechanical cleaning process. The dashed circle is the field of view of the STEM imaging shown in panel (**f**). **f** HAADF-STEM image of the partially cleaned graphene sample

Figure 2a shows a graphene flake transferred onto the PDMS film. The graphene flake on the PDMS film was positioned onto the Si_3_N_4_ membrane region in the TEM grid and physical contact was established between the flake and membrane. After the release, the graphene flake was transferred onto the TEM gird as shown in Fig. 2b. Figure 2c demonstrates a close-up view of the optical microscope image. We then conducted AFM imaging of the graphene flake, which identified the suspended region as shown in Fig. 2d. We used a hole near the graphene flake’s edge, from which we were able to easily find the same location for subsequent TEM investigations.

We mechanically cleaned the graphene surface by contact-mode AFM scanning. We conducted the contact-mode scanning using a rectangular sweeping region, which is shown as the dashed rectangle in Fig. 2d. To directly investigate the efficiency of mechanical cleaning, we intentionally left some suspended sample areas uncleaned. After the contact-mode sweeping, we reobtained a topographic image of the sample surface using the non-contact AFM mode. The surface residues accumulated at the rectangular boundary, confirming that AFM-based scanning indeed mechanically displaced the surface residues.

The sample area cleaned with AFM was investigated via TEM characterizations. Figure 2f shows a high-angle annular dark-field (HAADF) STEM image of the hole presented in Fig. 2e. We clearly observed the accumulated residues, which formed a line on the left part of the image (Fig. 2f). The regions on the left and right sides across the residue line had distinct contrast under STEM mode. The right side had darker contrast with less residue coverage than the left-side region, indicating that mechanical cleaning was indeed achieved.

**Fig. 3:**
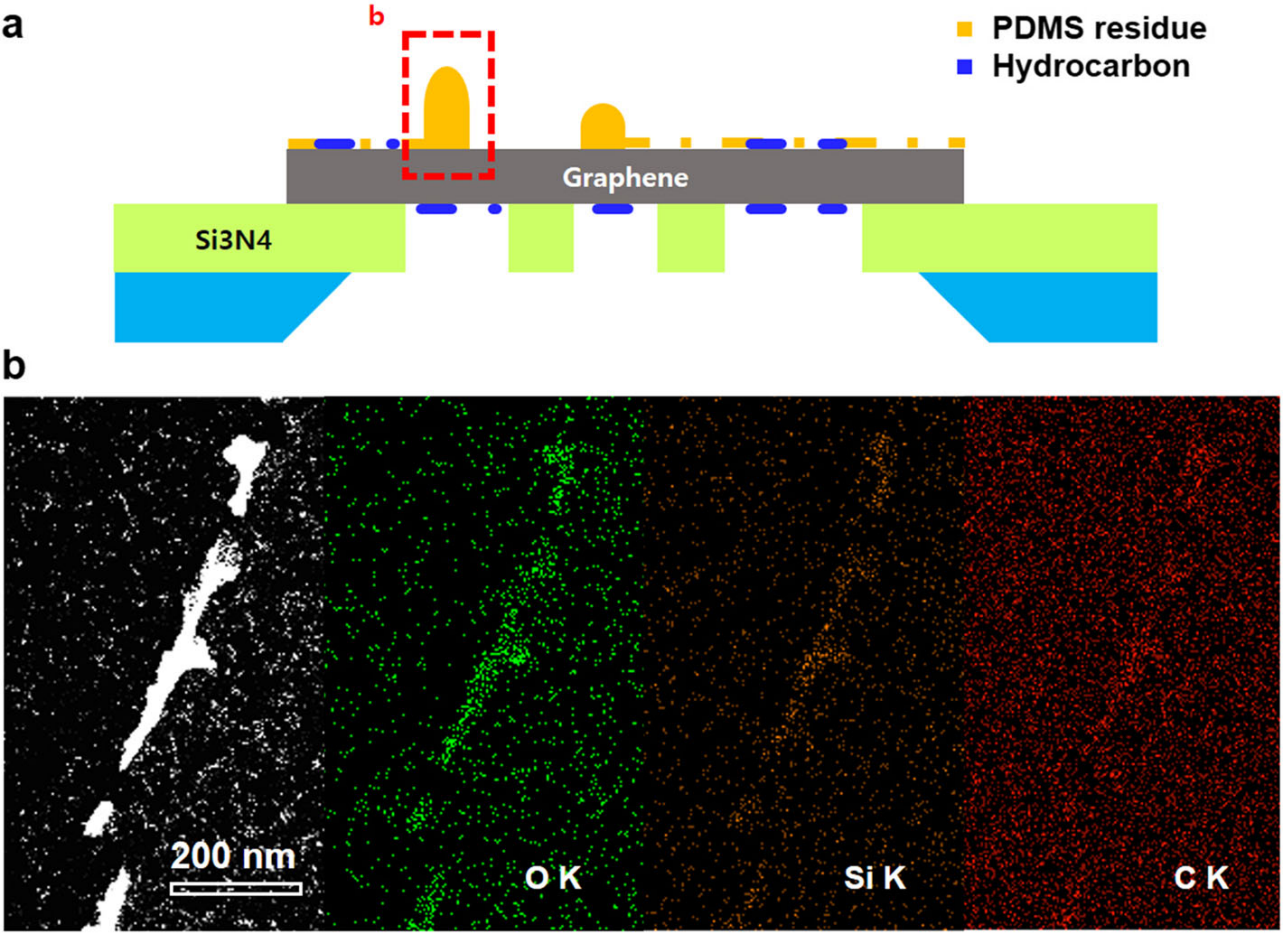
EDX investigation of residue aggregation induced by mechanical sweeping. **a** Schematic side view showing PDMS residue aggregated by mechanical sweeping. **b** EDX mapping around the residue aggregation. HAADF-STEM, oxygen K edge, silicon K edge, and carbon K edge mapping images are shown from left to right

Using EDX mapping, we analyzed the residues accumulated by AFM scanning as shown in Fig. 3. Figure 3b presents the HAADF-STEM image, oxygen K edge, silicon K edge, and carbon K edge intensity mapping data, respectively. Increased oxygen, silicon, and carbon signals occurred at the accumulated residue. The observed data were consistent with our interpretation that the surface residue was mainly PDMS accumulation (Fig. 3a); PDMS is composed of silicon, carbon, oxygen, and hydrogen.

**Fig. 4:**
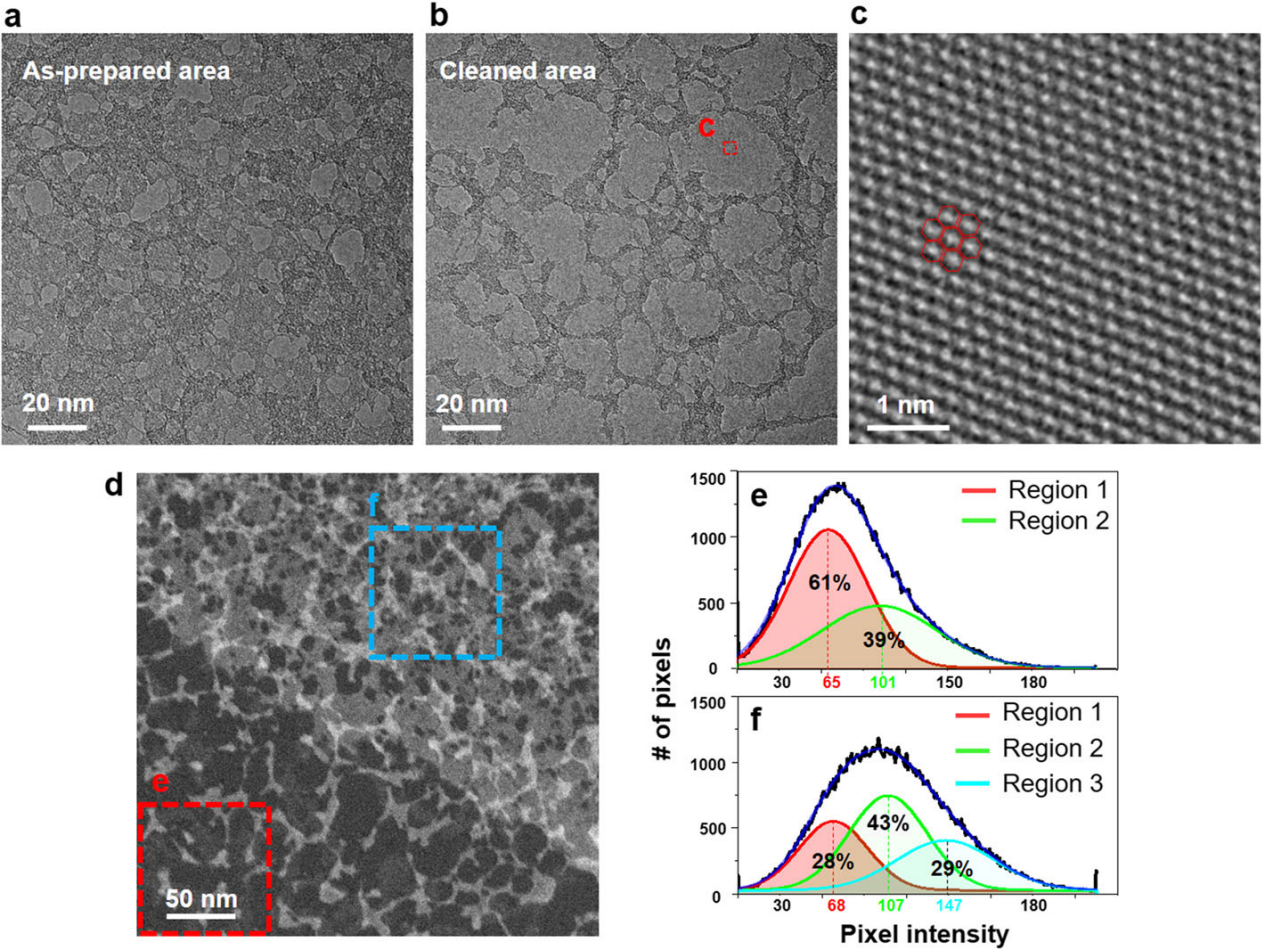
Residue coverage comparison between the as-prepared and mechanically cleaned area. **a** TEM image of the as-prepared graphene region. **b** TEM image of the mechanically cleaned graphene region. The red box is the field of view of panel (**c**). **c** Close-up TEM image showing the pristine graphene lattice without surface residue. The marked red hexagons represent the honeycomb lattice of graphene. **d** HAADF-STEM image of the partially cleaned graphene sample. The dark bottom region is from the mechanically cleaned region. The images in the dashed two boxes were used for the intensity analysis shown in panels (**e**) and (**f**). **e** Histogram of the pixel intensities shown in box e. The two peaks from relatively high- and low-intensity regions were deconvoluted from the histogram. **f** Histogram of the pixel intensities shown in box f. The three sub-peaks were deconvoluted from the histogram

We quantitatively investigated the effect of mechanical cleaning using TEM and STEM imaging as shown in Fig. 4. The as-prepared region without mechanical cleaning had typical residue networks as demonstrated in Fig. 4a. The individual residue-free region was approximately 10 nm wide. However, the mechanically cleaned region had a larger residue-free region that sometimes spanned an area larger than 20 nm. The close-up high-resolution TEM image clearly revealed a graphene lattice structure, demonstrating a pristine surface without residue (Fig. 4c).

STEM is more effective than TEM imaging to analyze the coverage and thickness of residues. The HAADF-STEM image demonstrated the clear contrast between the mechanically cleaned and as-prepared regions as shown in Fig. 4d. As expected, the mechanically cleaned region (bottom half, left) had darker contrast than the uncleaned region (top half, right). We plotted a histogram of the pixel intensity values and compared the two regions (dashed box in e and f). The cleaned region had a broad distribution and the maximum population was located at a mean pixel intensity of approximately 70. Based on the local pixel intensity, we identified two distinct contrast regions and deconvoluted the histogram as shown in Fig. 4e. The cleanest region (region 1) with a mean pixel intensity of 65 comprised approximately 61% of the sample area, and the relatively high-contrast region (region 2, with a mean pixel intensity of 101) comprised 39%.

The similar deconvolution process was applied to the histogram of pixel intensity data for the as-prepared graphene region (Fig. 4f). The STEM image of the as-prepared graphene region (Fig. 4d) displays mainly three distinct contrast regions. Based on this observation, the intensity histogram of the as-prepared graphene region was deconvoluted into three peaks (Fig. 4f). The cleanest region (region 1) with mean pixel intensity of 68 shared 28% of the sample area. This confirmed that the residue-free area more than doubled via mechanical cleaning. The regions with higher local intensities shared 43% and 29% for region 2 and regions 3, respectively. Because the mechanical cleaning process was performed only on side of graphene, the observed residue on the mechanically-cleaned graphene region is mainly adsorbed on the untreated side of graphene as shown in Fig. 3a. On the other hand, the residues on the as-prepared graphene region can be located on both sides (top and bottom surfaces) of graphene. Therefore, the deconvoluted peaks could be assigned as the regions with residue presence on one side (region 2) and on both side (region 3) of graphene, which is consistent with the observed local intensity pattern under STEM.

## Conclusion

In summary, we investigated the effect of mechanical removal of surface residues from graphene using various TEM-based characterizations. The mechanical cleaning process doubled the residue-free area compared to the uncleaned region, rendering more than 60% of the area without any surface residues. The residue-free region was directly confirmed with high-resolution TEM imaging, which clearly revealed the pristine graphene lattice structure. AFM-based mechanical cleaning is effective and applicable for preparing high-quality 2D crystals for atomic-resolution TEM investigations.

## Data Availability

The datasets used and/or analyzed during this study are available from the corresponding author on reasonable request.

## Abbreviations

2D: Two-dimensional
AFM: Atomic force microscopy
TEM: Transmission electron microscopy
PDMS: Polydimethylsiloxane
HAADF: High-angle annular dark field
STEM: Scanning transmission electron microscopy
EDX: Energy-dispersive X-ray spectroscopy
